# Current News

**Published:** 1901-07

**Authors:** 


### Current News.

### THE NATIONAL MEETINGS AT MILWAUKEE.—SPECIAL NOTICE.

THE profession from the Eastern section who expect to attend any or all of the national meetings at Milwaukee in August are requested to at once send their names to Dr. H. J. Burkhart, of Batavia, N. Y. It is expected that a rate war between the railroads will be on at that time, of which advantage will be taken. An arrangement can be made for a stop of several days at the Pan-American Exposition, at Buffalo, on the return trip.

### NATIONAL DENTAL ASSOCIATION.

The fourth annual meeting of the National Dental Association will be held in Milwaukee, Wis., commencing Tuesday, August 6, and continuing four days. The Masonic Temple hall, which is conveniently located and especially suited to the various needs of the Association, has been secured. Special railroad rates are being secured and will be announced later.

All regularly organized dental associations are entitled to one delegate for each ten members, and these associations are urged to send full delegations.

Dr. G. V. I. Brown, of Milwaukee, chairman of the local committee, will engage rooms at the hotels and answer questions regarding local arrangements.

G. V. Black,
*President.*
J. D. Patterson,
*Chairman Executive Committee.*
A. H. Peck,
*Secretary.*

---

### NATIONAL ASSOCIATION OF DENTAL EXAMINERS.

The nineteenth annual session of the National Association of Dental Examiners will be held at the Plankington Hotel, Milwaukee, Wis., beginning Friday, August 2, and continuing in session until adjournment. The hotel rates will be, American plan, without bath, $2.50 to $4.50 per day; with bath, $3.50 to $5 per day. A fare of one and one-third has been arranged for on the Lehigh Valley Railroad, good from July 31 to August 10, inclusive, $26.07 for the round trip. Trains leave New York at eight A.M., ten A.M., and twelve M.; leave Philadelphia, nine A.M., 10.30 A.M., and 12.30 P.M., connecting at South Bethlehem with main line; arrive at Buffalo, 9.20 P.M., 9.35 P.M., and 9.55 P.M., in time for connection with Chicago Express leaving at ten P.M.; arrive at Chicago next day, 1.28; leave Chicago at three P.M., and arrive at Milwaukee five P.M. Buy single through ticket and take agent's receipt.

Charles A. Meeker, D.D.S.,
*Secretary.*

